# Integrative analysis of cancer genes in a functional interactome

**DOI:** 10.1038/srep29228

**Published:** 2016-06-30

**Authors:** Matthew H. Ung, Chun-Chi Liu, Chao Cheng

**Affiliations:** 1 Department of Molecular and Systems Biology, Geisel School of Medicine at Dartmouth, Hanover, New Hampshire, 03755 USA; 2 Program in Quantitative Biomedical Sciences, Geisel School of Medicine at Dartmouth, Lebanon, New Hampshire, 03755 USA; 3 Institute of Genomics and Bioinformatics, National Chung Hsing University, Taiwan; 4 Norris Cotton Cancer Center, Geisel School of Medicine at Dartmouth, Lebanon, New Hampshire, 03766 USA

## Abstract

The post-genomic era has resulted in the accumulation of high-throughput cancer data from a vast array of genomic technologies including next-generation sequencing and microarray. As such, the large amounts of germline variant and somatic mutation data that have been generated from GWAS and sequencing projects, respectively, show great promise in providing a systems-level view of these genetic aberrations. In this study, we analyze publicly available GWAS, somatic mutation, and drug target data derived from large databanks using a network-based approach that incorporates directed edge information under a randomized network hypothesis testing procedure. We show that these three classes of disease-associated nodes exhibit non-random topological characteristics in the context of a functional interactome. Specifically, we show that drug targets tend to lie upstream of somatic mutations and disease susceptibility germline variants. In addition, we introduce a new approach to measuring hierarchy between drug targets, somatic mutants, and disease susceptibility genes by utilizing directionality and path length information. Overall, our results provide new insight into the intrinsic relationships between these node classes that broaden our understanding of cancer. In addition, our results align with current knowledge on the therapeutic actionability of GWAS and somatic mutant nodes, while demonstrating relationships between node classes from a global network perspective.

In recent decades, a plethora of high-throughput cancer genomic data has been generated in an attempt to better understand tumorigenesis and patient response to anti-cancer drug treatments. GWASs have identified germline variants associated with cancer predisposition and high-throughput sequencing projects have revealed several recurring mutations present in patient tumors. In lieu of these rich datasets, organized and integrated analyses are necessary to dissect the relationships between genetic predictors of cancer development and drug treatment response. In particular, identifying the relationships between germline variants, genes with recurrent somatic mutations, and drug targets can provide novel insight into the cancer interactome. Furthermore, carrying out analyses with a focus on drug targets can help translate basic genomic findings into the clinic by generating new hypotheses involving genes that can be targeted for therapy^1^. For example, Nelson *et al*. performed a large-scale study by integrating genomic and pharmacological data and found that genes previously identified to be associated with disease were significantly enriched in drug targets^2^.

One approach to studying disease genes and drug targets involves the use of networks to characterize systems-level relationships between genetic mediators of carcinogenesis^3^. Networks have been effective in identifying modules of interconnected genes that play important roles in a particular disease context^4^. Additionally, like social networks, distances between genes suggest high or low likelihood of co-activity^4^. Thus, networks remain useful tools for characterizing biology at a global level and for generating novel hypotheses that can be further tested experimentally^5^,^6^.

Despite the popularity of networks, there have been few studies that have applied network-based analysis to study potential relationships between cancer-related germline variants detected from genome-wide association studies (GWAS), somatic mutations found in tumors, and gene products targeted by known therapeutics. One study by Cao *et al*. utilized an undirected functional interaction network to analyze the relationships between drug targets and GWAS genes and found that GWAS genes were closer to drug targets than randomly chosen genes^7^. Sun *et al*. carried out a similar analysis focusing on 5 different disease groups including cancer, immune, metabolic, cardiovascular, and the nervous system^8^. However, these studies did not utilize directed edge information in their analysis and did not incorporate somatic mutation data.

Incorporating directionality allows for the dissection of relative distances between a pair of nodes and provides insight into the direction of information flow^9^,^10^. In the context of drug treatment, directionality provides information about downstream proteins that are activated by the drug target or vice versa, which is important for establishing a hierarchy. For example, if an oncogene is downstream of a drug target, we can deduce that it may not be the most effective target since its inhibition would not block the signal originating from the oncogene. However, if a drug target lies closely downstream of an oncogene then it could potentially halt or abrogate overactive signaling. Therefore, directionality allows us to determine if a node is actually blocking flow of information that travels one way. In addition to directionality, we also incorporate somatic mutation data, which in the context of cancer, have a higher likelihood of being functional and thus causal drivers of carcinogenesis compared to individual germline variants that are considered outside of a greater genetic interaction network. Thus, by combining these different node classes we can determine their relative positioning in directed pathways and evaluate topological differences between them that provide novel insight into how they may be affected by drug target inhibition.

Hence, in this analysis, we characterized the relative distances between cancer-associated GWAS gene nodes (GGNs), genes with known somatic mutations (SMNs), and drug targets (DTNs) in the context of a directed functional interactome. We find that these node classes exhibit non-random network topologies and non-random path characteristics. To our knowledge, this is the first study to analyze relationships between drug targets genes and disease genes using a permutation-based framework that incorporates information on edge directionality.

## Results

### Overview of analysis

**Figure 1:**
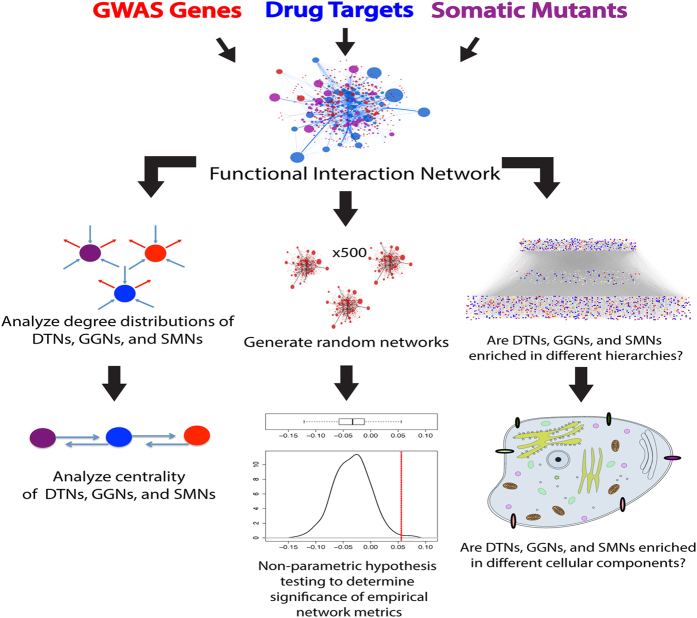
Flowchart of analysis. Three gene classes were derived from public databases and mapped to a functional interactome. 1. Network analysis of these gene classes were performed to identify topological relationships between these three node classes. 2. Accessibility between node classes was evaluated using a permutation based hypothesis testing framework where the geodesic ratio was calculated as the test statistic. 3. Hierarchical analysis was performed to identify hierarchical relationships between the node classes. Subsequent GO enrichment analysis was carried out to determine cellular localization of nodes.

Throughout our analysis, we utilized a functional interaction network published by Wu *et al*. that characterizes directed relationships between genes and gene products^11^. As a result, we do not restrict ourselves to purely protein-protein interaction or gene-gene interaction networks. To characterize the topological characteristics between GGNs, SMNs, and DTNs in the context of a directed functional interaction network, we computed their centrality using a variety of measures including degree, control, closeness, and betweenness. Second, we evaluated the extent to which sub-networks corresponding to each of these node classes exhibited scale-free properties. Third, to provide insight into the network distances between the three node classes, we calculated the relative reachability of each node class from and to another node class. If two node classes are considered for example, DTNs and GGNs, we start by calculating the number of existing geodesics (shortest paths between pairs of nodes) that exist from DTNs to GGNs and vice versa to determine if there are any differences in reachability from one node class to another. Of the geodesics that exist, we then calculated the average geodesic length from the first class to the second class, and vice versa to determine differences in network distance between two node classes. We further developed a simple metric we termed as the “geodesic ratio,” which compares the geodesic lengths of one node class to another to determine their relative accessibility. We then computed the statistical significance of each geodesic ratio by comparing the geodesic ratio to a null distribution of geodesics derived from degree-preserved random networks generated by edge permutation. Lastly, we calculated the enrichment of genes belonging to each node class in different network hierarchies and in different cellular components (Fig. 1).

### Shared genes between DTNs, GGNs, and SMNs

**Figure 2:**
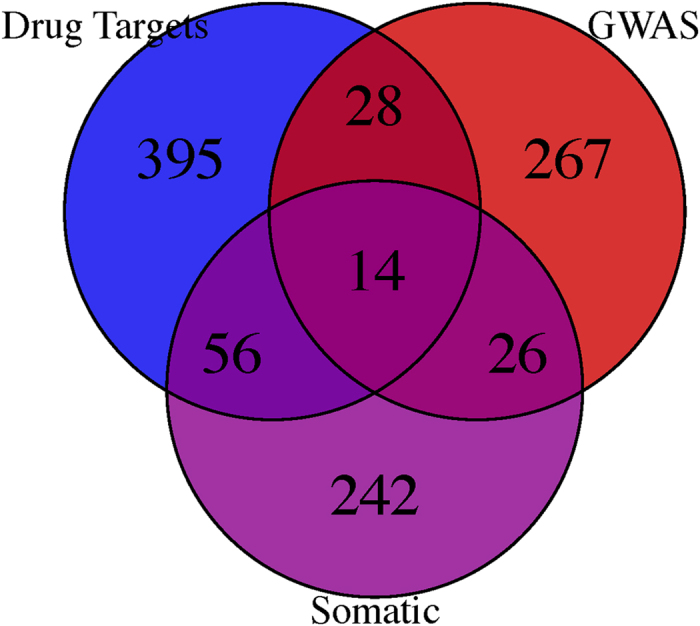
Venn diagram of node overlap. The number of genes belonging to each node class. The highest overlap was between DTNs and SMNs (n = 70) whereas the lowest overlap was between GGNs and SMNs (n = 40).

Several anticancer drugs function by inhibiting overactive oncogenic pathways that confer a proliferative advantage to neoplastic cells^12^. Therefore, we aimed to determine if any genes with identified germline or somatic mutations were also directly targeted by known drugs. As expected, we found that the highest overlap is 70 between DTNs and SMNs (P = 6.2E-18, OR = 4.0), compared to an overlap of 42 between GGNs and DTNs (P = 1.3E-4, OR = 2.0) and 40 between GGNs and SMNs (P = 3.0E-8, OR = 3.0) (Fig. 2). This supports the idea that many drug targets are somatic mutants since they are, in many cases, the drivers of carcinogenesis and are thus purposefully targeted by current therapeutics (e.g. PIK3CA)^13^,^14^,^15^.

### Characterizing the degree distribution of the interactome, DTNs, GGNs, and SMNs

**Figure 3:**
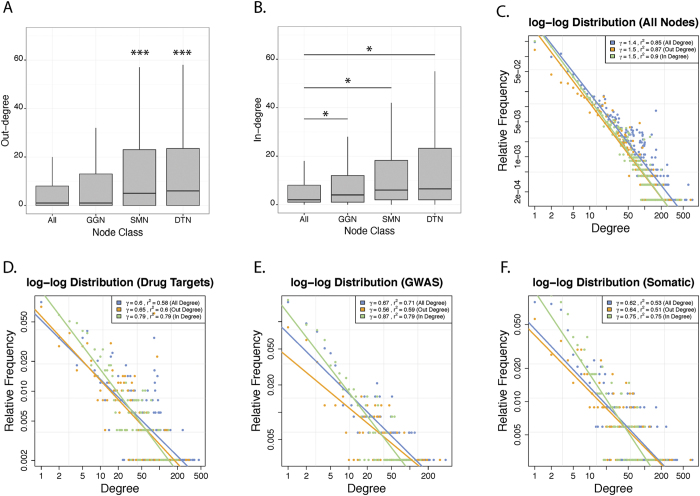
Degree distribution of node classes and identification of scale-free properties. (**A**) Out-degree distribution of all node classes compared to all nodes. ***indicates significant difference in out-degree with all other node classes at P < 0.01. (**B**) In-degree distribution of all node classes compared to all nodes. *indicates P < 0.01. (**C**) Log-log plot of the degree distribution (out-degree and in-degree) for all nodes. (**D**) Log-log plot of the degree distribution (out-degree and in-degree) for all nodes. (**E**) Log-log plot of the degree distribution (out-degree and in-degree) for all nodes. (**F**) Log-log plot of the degree distribution (out-degree and in-degree) for all nodes.

In the context of a network, it has been shown that disease genes tend to have higher degree centrality compared to random genes, indicating that their perturbation would result in greater widespread cellular changes due to their large number of connections^16^. To evaluate this hypothesis in a directed interactome, we first calculated the degree centrality of the three node classes. We found that DTNs and SMNs have the highest out-degree suggesting that they regulate many downstream pathways and thus exhibit greater importance in the regulation of cellular processes (Fig. 3A, P < 0.01, Wilcoxon test). However, GGNs do not have significantly higher out-degree compared to the entire interactome, suggesting that they provide limited information when considered on an individual basis and not within a larger polygenic architecture (Fig. 3A). This observation is in accordance with the failure of several genome-wide association studies to translate findings into the clinic^17^,^18^,^19^. Indeed, GGNs may be in linkage disequilibrium with causal drivers, and when considered in combination with other GGNs can reveal substantial information about their functions underlying carcinogenesis. Therefore, efforts to functionally annotate and identify interactions between GNNs has the potential to summarize GGNs more effectively.

Second, we observed that DTNs and SMNs also exhibit greater in-degree compared to the interactome (P < 1E-16, Wilcoxon test) but do not differ between each other (P = 0.9, Wilcoxon test). Additionally, GGNs also show greater in degree compared to the interactome (P = 0.003, Wilcoxon test). Overall, these results indicate that although DTNs and SMNs regulate many gene products, they are also subjected to either increased regulation or serve as “bottleneck” proteins as shown earlier. Furthermore, the fact that GGNs also exhibit higher in-degree but no difference in out-degree compared to the interactome suggests that it is still slightly more functionally relevant than background nodes.

Since we showed that the three node classes exhibit significant differences in degree distribution, we next aimed to evaluate the scale-free properties of the entire network and the subnetworks consisting of a single node class. Scale-free properties are a major hallmark of biological networks and indicate the presence of key biomolecular “hubs” that play major roles in the regulation of living systems^20^,^21^,^22^. First, by fitting a linear regression model with log-transformed node degrees as the predictors and log-transformed degree frequency as the response, we observed that the entire functional interaction network is scale-free with γ > 1 and r^2^ > 0.8 in terms of both out-degree and in-degree distributions (Fig. 3A). Furthermore, we observed that sub-networks formed by DTN, GGN, and SMN node classes also exhibit scale-free properties, indicating that there exists within-class “hubs” (Figs 3D–F and S1).

### GGNs are more accessible from DTNs

Distance between nodes in network space is another topological measure that provides information about the likelihood of two nodes interacting with each other in cellular space^4^. In the context of a directed functional interaction network, distance measures (geodesics) between node pairs presumably indicate hierarchical positions of nodes in a biological pathway, which informs us of their functional importance and potential druggability^23^,^24^,^25^,^26^. Therefore, a key aim of this study was to compare reachability and accessibility from one node class to another node class by utilizing the directed edge information contained in the functional interaction network. Here, we sought to analyze the topological relationships between GGNs and DTNs by characterizing the geodesics originating from DTNs to GGNs, and similarly from GGNs to DTNs.

**Figure 4:**
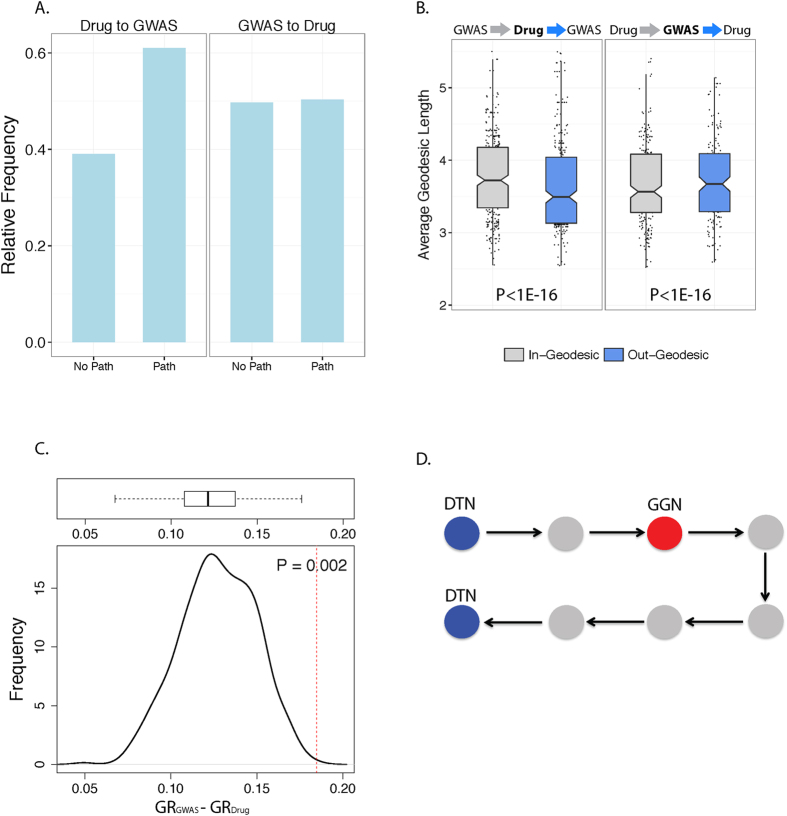
Analysis of geodesics between GGNs and DTNs to evaluate relative accessibility. (**A**) Fraction of existing and non-existing paths from DTNs to GGNs, and fraction of existing and non-existing paths from GGNs to DTNs. (**B**) Left: Distribution of mean geodesics from GGNs to DTNs, and distribution of mean geodesics from DTNs to GGNs in DTN-centric analysis. Each point represents the mean out/in geodesic of each GGN to all DTNs. Right: Distribution of mean geodesics from DTNs to GGNs, and distribution of mean geodesics from GGNs to DTNs in GGN-centric analysis. Each point represents the mean geodesic of each DTN to all GGNss. Note: *P-values were calculated based on all geodesics prior to taking the mean.* (**C**) Null distribution generated by calculating difference in geodesic ratios between GGNs and DTNs in 500 random edge-permuted networks. Red dotted line indicates the observed value of difference in geodesic ratios calculated from the empirical network. (**D**) Example diagram showing greater accessibility from a DTN to a GGN, compared to a GGN to a DTN.

First, reachability is a binary measure that indicates whether a path exists between any given node pair in a directed network. To determine reachability, we considered the fraction of geodesics that exist out of all possible paths between each GGN-DTN pair. We found that 60% of all possible geodesics exist from DTNs to GGNs compared to 40% that do not exist in the network (Fig. 4A). However, we observed that the fraction of geodesics that exist from GGN to DTNs is approximately equal to the fraction of geodesics that do not exist (Fig. 4A). These results suggest that GGNs are more reachable from DTNs, as opposed to the case where DTNs are more reachable from GGNs. However, these differences in reachability can be explained by differences in degree between DTNs and GGNs, such that reachability of a target node is correlated with the degree of the source node. To confirm, we generated 500 random degree-preserved networks to generate a null distribution of odds ratios and found that our observed odds ratio is not unexpected by chance (Fig. S2).

Second, we analyzed the accessibility of node classes by calculating the lengths of geodesics that exist between DTNs and GGNs. We adopted a DTN-centric and GGN-centric approach whereby we calculated the mean out- and in-geodesic length of each node class. In particular, for the DTN-centric analysis, we calculated the mean length of geodesics outgoing from DTNs to GGNs and the mean lengths of geodesics incoming from GGNs. Similarly, the same was done for the GGN-centric analysis. In the DTN-analysis, we observed that the mean geodesic length from drug nodes to GGNs was significantly less than that of geodesics incoming from GGNs (Fig. 4B). In the GGN-centric analysis, the geodesics outgoing from GGNs to DTNs tend to be longer than geodesics incoming from DTNs, which is consistent with the DTN-centric results (Fig. 4B). This indicates that GGNs are more accessible from DTNs suggesting that DTNs function upstream of GGNs.

Third, operating under the assumption that a gene product will be most affected by the nearest inhibited drug target and less affected by inhibition of distant drug targets, we reasoned that it is more informative to consider the mean geodesic length between GGNs and their nearest DTN (as opposed to the mean of all geodesics), and conversely between DTNs and their nearest GGN. Additionally, considering the ratio of mean outgoing geodesics to mean incoming geodesics for all nodes allows for a comparative measure of geodesic length. Thus, we used the difference between the geodesic ratios of each node class (See methods) as a test statistic by combining out-degree and in-degree geodesic lengths into a single ratio, and calculated the difference between the DTN geodesic ratio and the GGN geodesic ratio. Furthermore, we aimed to determine if the difference in geodesic ratios between two node classes was confounded by the inherent topological structure of the network (i.e. differences in degree centrality) caused by ascertainment bias, or truly exhibited non-random localization of nodes. To address this, we permuted the edges of our functional interaction network to generate 500 random degree-preserved networks and recalculated the difference in geodesic ratios in each random network to obtain a null distribution of difference in geodesic ratios. Using this non-parametric significance testing approach, we observed that the difference in geodesic ratios between drug target genes and GGNs was >1 and significant even when node degree was preserved (P = 0.002, Fig. 4C). This indicates that GGNs are more accessible from DTNs, suggesting that drug targets exhibit greater functional influence over genes with identified germline variants (Fig. 4D). For example, if the activity of drug targets were to be altered, downstream germline variants will be affected at some level. However, in the case were the activity of germline variants were altered, the effect on drug targets may not be as substantial since signal propagation will be slower due to longer paths to drug targets. Overall, these results suggest that gene products with identified cancer-associated germline variants tend to localize downstream of the functional pathway of drug target gene products (See discussion).

### SMNs are more accessible from DTNs

**Figure 5:**
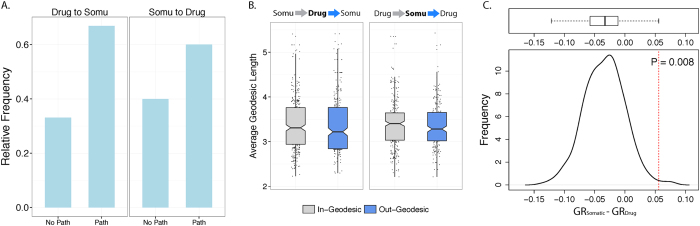
Analysis of geodesics between SMNs and DTNs to evaluate relative accessibility. (**A**) Fraction of existing and non-existing paths from DTNs to SMNs, and fraction of existing and non-existing paths from SMNs to DTNs. (**B**) Distribution of mean geodesics from SMNs to DTNs, and distribution of mean geodesics from DTNs to SMNs in DTN-centric analysis. Each point represents the mean out/in geodesic of each SMN to all DTNs. Right: Distribution of mean geodesics from DTNs to SMNs, and distribution of mean geodesics from SMNs to DTNs in SMN-centric analysis. Each point represents the mean out/in geodesic of each DTN to all SMNs. Note: *P-values were calculated based on all geodesics prior to taking the mean.* (**C**) Null distribution generated by calculating difference in geodesic ratios between SMNs and DTNs in 500 random edge-permuted networks. Red dotted line indicates the observed value of difference in geodesic ratios calculated from the empirical network.

Because cancer-associated somatic mutants have been identified to be involved in carcinogenesis, we hypothesized they would exhibit similar functional influence as drug targets. In support of this, we observed that the reachability of DTNs to SMNs is similar to the reachability of SMNs to DTNs (Fig. 5A). Similarly, from a DTN-centric analysis, the average length of out-degree geodesics to SMNs is non-significantly shorter than the average length of in-degree geodesics from SMNs (Fig. 5B). The same pattern is observed from a SMN-centric analysis (Fig. 5B). However, we observed a significant difference in geodesic ratios between DTNs and SMNs as calculated from the null distribution of geodesic ratio differences generated from degree-preserved random networks (P = 0.008, Fig. 5C).

These results indicate that there is no significant difference in relative reachability between DTNs and SMNs and no difference in relative average length of existing geodesics between the two node classes. However, the difference between the geodesic ratios of the two node classes is significant suggesting that drug targets tend to be upstream when nearest node pairs are considered. Additionally, this provides support for our reasoning that analyzing the shortest geodesics between each pair of nodes can reveal the regulatory or functional hierarchy between two node classes that, otherwise, cannot be detected by taking the global average of geodesic lengths.

### DTNs, SMNs, and GGNs exhibit differences in control centrality, closeness centrality, and information flow

**Figure 6:**
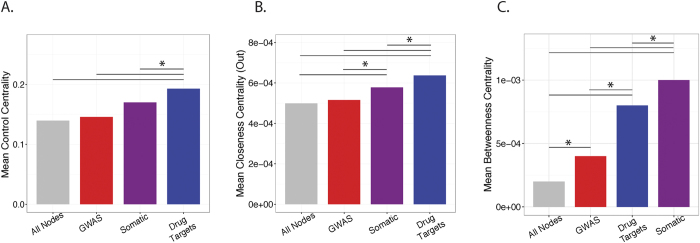
Centrality measures of DTNs, SMNs, and GGNs. (**A**) Barplot comparing normalized mean control centrality between DTNs, SMNs, and GGNs. (**B**) Barplot comparing normalized out-closeness centrality between DTNs, SMNs, and GGNs. (**C**) Barplot comparing mean out-betweenness centrality between DTNs, SMNs, and GGNs. (*P < 0.01)

To complement our previous analysis, we performed controllability analysis to compare the three node classes in terms of their network importance. The controllability of a network refers to the ability to drive a network from an initial state to any desired final state by controlling the activity of a subset of nodes^27^. Intuitively, nodes that are important for driving a network to a different state exhibit high control centrality^27^. When comparing the control centrality of our node classes, we found that DTNs had significantly higher control centrality than SMNs (P = 6E-3) or GGNs (P = 1.6E-5) (Fig. 6A). These results are consistent with our finding that SMNs and GGNs are more accessible from DTNs. Controllability analysis indicates that DTNs exert greater control over the entire network which contributes to why there is evidence of their inhibition having therapeutic indications. Additionally, we compared our node classes in terms of their closeness centrality (out) and found a similar trend where where DTNs exhibited highest closeness centrality followed by SMNs and GGNs, respectively (Fig. 6B).

Furthermore, to evaluate the role of GGNs, SMNs, and DTNs in information propagation, we calculated the betweenness centrality of these node classes. Nodes with high betweenness centrality are known as “bottleneck” proteins due to their role in maintaining communication and signaling in biochemical and regulatory pathways^9^,^28^,^29^,^30^. We observed that SMNs exhibit the largest average betweenness centrality followed by DTNs, GGNs, and all nodes combined, respectively (Fig. 6C). Moreover, the differences in betweenness centrality between all node classes were all statistically significant (P < 0.01, Wilcoxon test, Fig. 6C). These results suggest that genes with recurrent mutations in cancer (SMNs) are play a role in connecting together different biological pathways. As such, it is no surprise that somatic mutations in these genes are detected in cancer since they tend to play essential roles in maintaining cellular homeostasis and genomic fidelity^31^,^32^,^33^,^34^. In the case of DTNs, it seems reasonable that drugs may not always be targeted to genes containing somatic mutations, but instead are targeted to genes that may contribute to the progression of the disease due to their involvement in the same pathway or in a complementary pathway^35^,^36^,^37^. These genes are functionally important and may modulate or amplify cancer-associated pathways driven by SMNs, and may also represent alternative targets that are more therapeutically actionable from a practical perspective^38^. Furthermore, the average betweenness centrality of GGNs are consistent with the established notion that although genes identified from GWAS studies may be informative to cancer-related predispositions, they may not themselves be causal factors^39^.

### DTNs, GGNs, and SMNs are enriched in different network hierarchies and biological components

**Figure 7:**
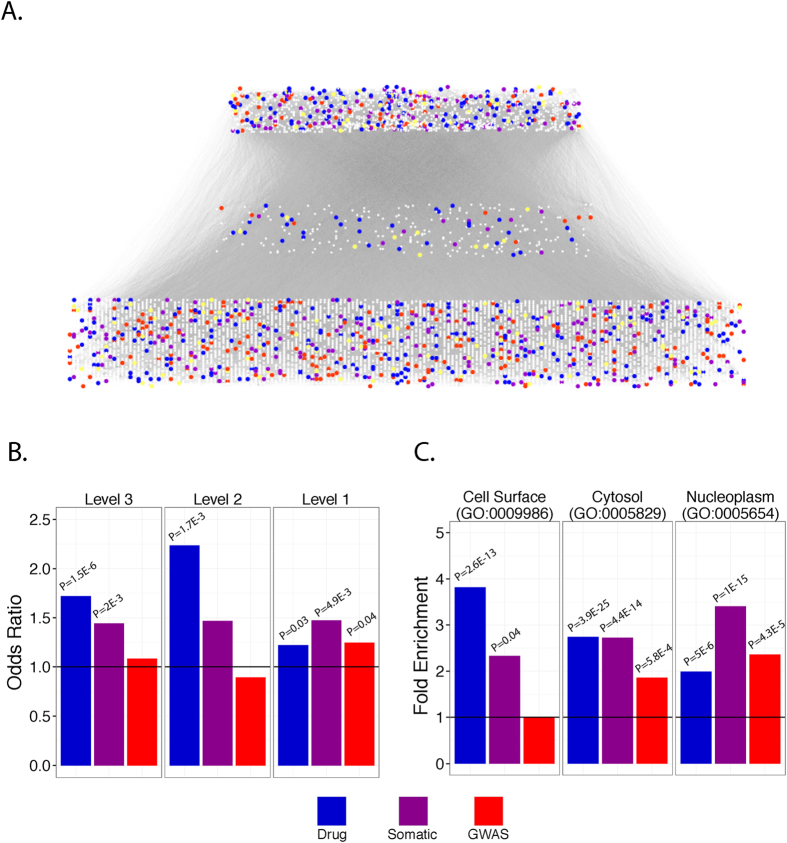
Enrichment of node classes in network hierarchies. (**A**) Odds ratios for the enrichment of each node class in high, middle, and lower network hierarchies. Bold horizontal line corresponds to an odds ratio of 1 indicating no enrichment. (**B**) Fold enrichment of GO:0009986 (Cell Surface), GO:0005829 (Cytosol), and GO:0005654 (Nucleoplasm) in each node class. *Adjusted P≤0.05. (**C**) Functional interactome organized into top, middle, and lower hierarchies.

Typically, biological networks are composed of biomolecules that play different roles, some major and some minor. Naturally, it is expected that dominant regulators integral to cellular homeostasis or survival would be classified as higher order nodes. As such, we applied the hierarchical score maximization algorithm (HSM) to stratify the network into three hierarchical layers: top, middle, and bottom (Fig. 7A)^40^. We then calculated the enrichment of each node class in each hierarchical layer. In the top layer, we observed that DTNs were enriched 2.01-fold (P = 6E-13), SMNs were enriched 1.55-fold (P = 2E-4), and GGNs were enriched 1.30-fold (P = 0.03). In the middle layer, we found that only SMNs were significantly enriched 1.36-fold (P = 0.03). In the bottom layer, we found that only GGNs were enriched 1.29-fold (P = 0.03) (Fig. 7B). These results suggest that DTNs and SMNs are more functionally more important compared to GGNs, which are enriched primarily in the bottom network layer.

To further characterize DTN, GGN, and SMN sets we performed Gene Ontology (GO) enrichment analysis to determine which cellular components they are enriched in. We found that DTNs were most enriched in Cell Surface (GO:0009986) (3.82-fold, P = 2.6E-13). Additionally, DTNs were also enriched 2.74-fold and 2-fold enriched in Cytosol (P = 3.9E-25) and Nucleoplasm (P = 5E-6), respectively. In contrast, SMNs were enriched 3.4-fold in Nucleoplasm (1E-15), 2.7-fold in Cytosol (4.4E-14), and 2.3-fold in Cell Surface (P = 0.04). GGNs were significantly enriched 2.4-fold in Nucleoplasm (P = 4.3E-5), 1.94-fold in Cytosol (P = 5.8E-4), and was not significantly enriched in Cell Surface (Fig. 7C). This indicates that GGNs, SMNs and DTNs are localized in different cellular components. These results combined with our observation that DTNs act as “hub” proteins while SMNs act as “bottleneck” proteins^28^,^29^,^30^, led us to speculate that drug targets were more likely to be membrane receptors that initiate signaling cascades, which propagate through somatic mutants in the cytoplasm, which then subsequently induce transcription of GWAS genes in the nucleus.

## Discussion

In this analysis, we introduce a network-based approach to study the relationships between cancer-associated drug targets, germline variants, and somatic variants in the context of a directed functional interactome. We introduce a new approach to analyzing the geodesics between the three node classes by designing a geodesic ratio and implementing a non-parametric approach to evaluate significance. Furthermore, we show that different node classes are enriched in different hierarchical layers and the results are consistent with the geodesic-based results (Figs 4 and 5, S3). Lastly, we show that the different node classes are enriched in different biological components. Our results suggest a functional hierarchy whereby drug targets exhibit greatest functional influence, followed by somatic mutants and germline variants, respectively. We also note that germline variants are still under intense investigation to functionally annotate them and summarize the information within combinations of variants. Analysis of germline variants in the context of a greater genetic interaction network may identify genes that exhibit higher functional hierarchy compared to individual variants used in this analysis.

Since this study involved geodesics between different node classes, it draws upon the concept of network parsimony whereby two nodes that are closer together in a biological network are more likely to be involved in a functional relationship. In directed walks, if geodesics from source nodes are shorter than the geodesics from target nodes to the source nodes, the source nodes presumably exert greater functional influence over the target nodes (Fig. 4D). For example, if source nodes were to be perturbed, the effect on target nodes would be greater than the effect on source nodes if target nodes were to be perturbed instead.

Interestingly, we observed that SMNs have significantly greater betweeness centrality compared to DTNs suggesting that they function more as “bottleneck” proteins^28^,^29^,^30^. On the other hand, DTNs have greater degree centrality indicating that they are “hubs” in the functional interactome. This is further supported by the observation that DTNs are enriched on the cell surface and SMNs are enriched in the cytoplasm. Since SMNs tend to involve signaling kinases that function in cell proliferation, anti-apoptosis, and survival pathways, it is reasonable that they function as “bottleneck” proteins that transfer signals from DTNs to genes in the nucleus. Furthermore, the hierarchical differences revealed by geodesic analysis of DTNs and SMNs are not as pronounced as those between DTNs and GGNs. This suggests that they are highly intertwined in the regulation of each other. Finally, the large overlap between DTNs and SMNs compared to the smaller overlap between DTNs and GGNs indicate that SMNs tend to be the drivers of oncogenesis since they are targeted therapeutically.

Our results also provide insight into the state of drug treatment. We speculate that drugs targeting SMNs or gene products directly downstream of SMNs would block signaling throughout the cascade and thus be more efficacious^13^. Since several somatic mutations result in constitutively active signaling, it may not be the best strategy to target proteins upstream of the mutant protein^41^. The observation that DTNs have a tendency to lie upstream of SMNs, led us to suspect that the current repertoire of available anticancer agents cannot yet target the proper proteins.

We acknowledge that there exist limitations associated with this study. First, cancer is a collection of separate diseases, each with different underlying causes. In this analysis, we study the three node classes from a global view of cancer to identify patterns that may yield general properties between these node classes in all cancers. As a result, some cancers may not exhibit the trends presented in this study due to differences in the underlying genomic architecture. Second, biological interpretation of a geodesic in the context of a functional interactome is limited in that each geodesic may represent a different biological relationship between two nodes that requires case-by-case analysis to fully understand its implications.

A key obstacle encountered in this study involves the fact that biological networks are prone to false positive interactions and incompleteness due to lack of comprehensive experimental data upon which networks are constructed from. Furthermore, the functional interaction network used in this study was derived from a collection of knowledge-based pathway databases containing high confident annotations (Reactome, Panther, CellMap, NCI, KEGG, TRED). In this analysis, we favored high-confident interactions at the cost of high coverage to preserve specificity. This incompleteness may introduce ascertainment bias in the functional interaction network whereby protein-protein or protein-gene interactions that are under more intense study in the literature are overrepresented in the network. Conversely, biological interactions that exist but are not yet defined will be absent from the network. To address this issue, we have taken necessary steps to mitigate the effects caused by network incompleteness and ascertainment bias. Specifically, we adopted a degree-preserved edge permutation approach to generate null networks that we use to compare our empirical results. By randomly assigning each node a new set of interactions while maintain its degree, we effectively create a null network model that takes these degree biases into consideration. Furthermore, other studies have applied these network permutation methods to reduce bias when studying PPI networks associated with immune and cancer related diseases^42^,^43^.

We maintain that our study provides novel insight into the relationship between drug targets, germline variants and somatic mutations by using a directed functional network. We utilized comprehensive databases and analyzed the relationships between the three node classes using a variety of approaches. Additionally, our analysis is focused on dissecting the hierarchical relationships between three types of information and found that DTNs tend to lie upstream of the functional hierarchy followed by SMNs, and finally GGNs. These results are consistent with the concept of “rational drug design” where several drug targets were identified based on their recurrence in tumor biopsies as in the case of c-Met inhibitors^44^. Additionally, there is extensive ongoing research into methods that can extract more therapeutically actionable information from germline variants by analyzing them in combination instead of individually^45^. To our knowledge, there has been no other study that has systematically integrated these different classes of data and dissected their positional placement using network directionality information. Overall, this study provides novel insight into the hierarchical ordering of three node classes, which may reflect intrinsic properties of human interactomes that are associated with carcinogenesis. An investigation that accounts for directionality is critical in therapy because the relative positions of cancer drivers dictate where in the signaling pathway a drug should inhibit. Furthermore, we applied two different methodologies to investigate this hierarchy including the use of the geodesic ratio and the hierarchical score maximization algorithm.

## Materials and Methods

### Data procurement

Annotated Version 2014 of the functional interactome was downloaded from the Reactome website http://www.reactome.org/pages/download-data/46. The network consisted of interactions derived from REACTOME, Panther, CellMap, NCI Pathway Interaction Database, KEGG, and TRED^46^,^47^,^48^. The network included functional protein interactions and TF-gene interactions, along with computationally predicted interactions^11^. Predicted edges were removed to only include confident interactions and gene symbols that did not match known Ensembl genes were removed. The final network consisted of 7222 nodes and 83404 directed edges.

GWAS data was downloaded from the NHGRI-EBI GWAS catalog (http://www.ebi.ac.uk/gwas/). Genes that were associated with disease traits containing the terms: “cancer,” “neoplasm,” “tumor,” ”tumour,” “tumorigenesis,” “carcinogenesis,” or “carcinoma” with P < 1E-8 were extracted from the catalog. GWAS genes were further filtered to include only genes that were also in the network to yield a final set of 335 GGNs. Somatic mutation data was downloaded from the COSMIC database (http://cancer.sanger.ac.uk/cosmic) and filtered to include genes that were also in the network to yield a final set of 338 SMNs^49^. Drug targets were derived from the DGIdb database (http://dgidb.genome.wustl.edu/), which combines high confident drug target information from several sources including DrugBank, Cancer Commons, Clearity Foundation, My Cancer Genome, PharmGKB, and other publications^50^,^51^,^52^. These databases include information about known interactions between existing drugs and the proteins that they target, and what disease they are associated with. Cancer-associated drug targets identified in DrugBank and PharmGKB were filtered out based on their association with the following disease terms: “cancer,” “neoplasm,” “tumor,” ”tumour,” “tumorigenesis,” “carcinogenesis,” or “carcinoma”. The final set included 395 cancer-associated drug targets.

### Calculation of degree, control centrality, closeness centrality, betweenness centrality, and geodesics

Degree of node *v* is defined as the number of edges (out-degree, in-degree, or both) connected to *v.* One method to evaluate scale-free properties of a network is to generate log-log plots where a linear regression model is fitted to log-transformed degree distribution data. The r^2^ was calculated to determine goodness-of-fit of the linear model.

Scale-free networks have a degree distribution that follows the power law where


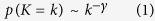


where *K* is node degree. To determine if the network is scale-free the log of the function is log-transformed to yield


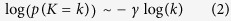


The coefficient of the model, also known as the degree exponent γ, describes the shape of the distribution.

Control centrality of all nodes were calculated using the “CalControlCentrality” program published by Liu *et al*.^27^. Closeness centrality is defined as:


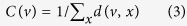


where *v* is the node of interest and *d*(*v, x*) is the distance from node *v* to the *x*_*th*_ connected node. Betweenness centrality is defined as:


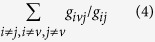


where *g*_*ij*_ is the total number of geodesics from node *i* to node *j,* and *g*_*ivj*_ is the number of geodesics from node *i* to node *j* that passes through node *v*. Calculation of betweenness was implemented using the algorithm proposed by Brandes^53^. Geodesics between all vertices were calculated using the breadth first search algorithm after removing all self-loops. Degree, closeness betweenness, and geodesics were calculated using the “igraph” R package.

### Calculation of geodesic ratio and significance testing

Prior to calculation of geodesic ratios, all self-loops (overlapping node classes) were removed to avoid geodesics of length 0 that may skew the ratio. The numerator of the geodesic ratio was calculated by considering the mean of all out-degree geodesics to the nearest target node (of the target node class). The denominator of the geodesic ratio was calculated by considering the mean of all in-degree geodesics from the nearest target node (of the target node class). The geodesic ratio can be represented using the formulas:


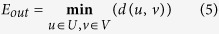



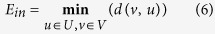



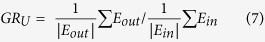


where U is the vector of all nodes within a node class, 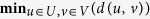 is the shortest of all out-degree geodesics from node *u* to all nodes in class *V,* and 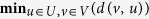 is the shortest of all in-degree geodesics from all nodes in class *V* to node *u. E*_*out/in*_ is a vector containing the shortest path between each *u*,*v* pair. The difference in geodesic ratio between two node classes was calculated (i.e. *GR*_*GGN*_−*GR*_*DTN*_) to yield a test statistic. A test statistic >1 indicates that the first node class is more accessible from the second node class and conversely, a test statistic <1 indicates that the second node class is more accessible form the first node class. To determine significance, 500 random degree-preserved networks were generated by edge permutation, and the test statistic was re-calculated for each network to yield a null distribution. The non-parametric p-value was calculated as the fraction of random networks that yielded test statistic greater than or equal to the observed test statistic.

### Hierarchical and biological component enrichment analysis

Nodes were assigned to one of three hierarchical classes with a given probability using the HMS algorithm^40^. The algorithm outputs the probability of a node belonging to each of the three hierarchical layers. Nodes were assigned to a layer if the probability of it belonging to the layer was >0.5. Fisher’s exact test was used to calculate enrichment of each node class in the top, middle, and bottom hierarchical levels. Network visualization was created using Cytoscape^54^. GO enrichment of the three node classes was implemented using the DAVID bioinformatics webtool (https://david.ncifcrf.gov/)^55^.

## Additional Information

**How to cite this article**: Ung, M. H. *et al*. Integrative analysis of cancer genes in a functional interactome. *Sci. Rep.* **6**, 29228; doi: 10.1038/srep29228 (2016).

## Supplementary Material

Supplementary Information
