# Supplementary material for: Integrative analysis of cancer genes in a functional interactome
Source: Sci Rep. 2016 Jun 30;6:29228. doi: 10.1038/srep29228 (PMC4928112; doi:10.1038/srep29228)
Supplement: Supplementary Information [file srep29228-s1.pdf]

Supplementary Information:

Integrative analysis of cancer genes in a functional interactome

Matthew H. Ung[1,2], Chun-Chi Liu[3], Chao Cheng[1,2,4,*]

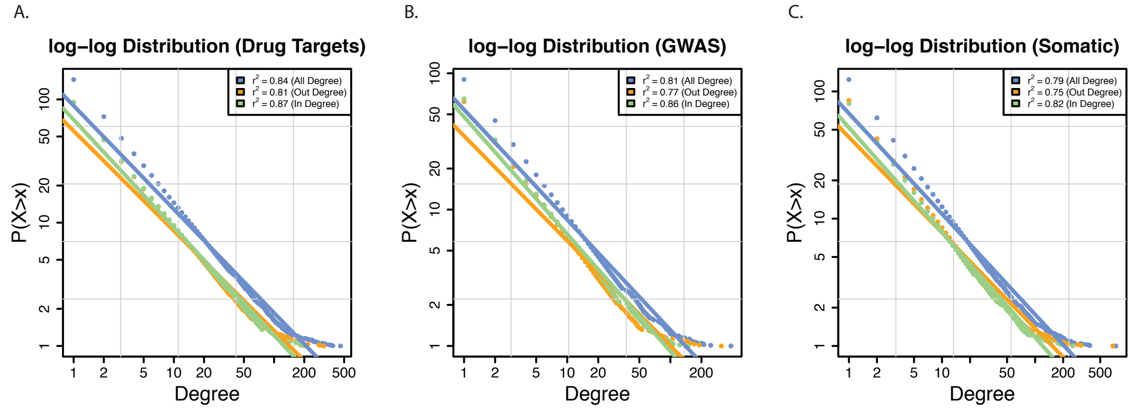

Figure S1. Degree distribution analysis of node classes (log-cumulative distribution). Double log-plot of node degree and cumulative node distribution for a) DTNs, b) GGNs, and c) SMNs.

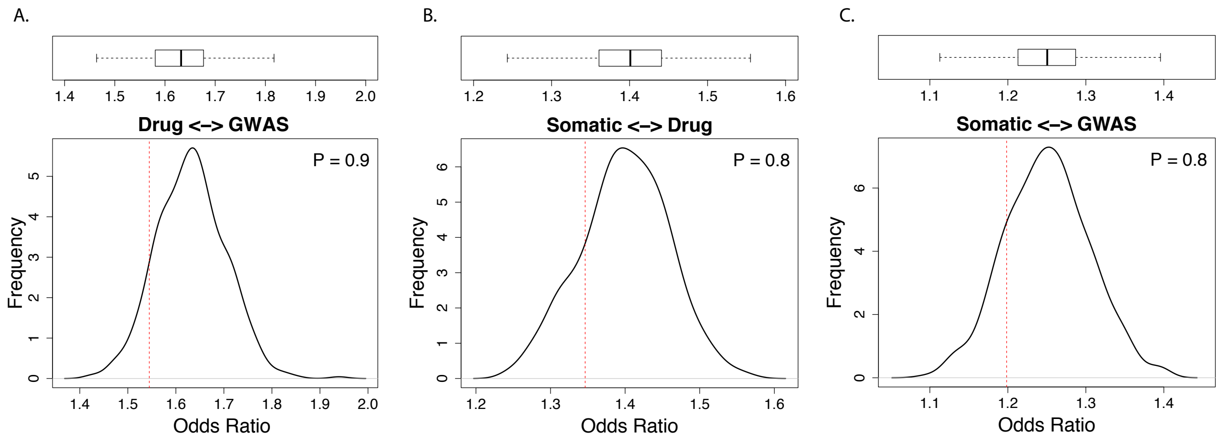

Figure S2. Null distribution of odds ratios. Odds ratios were calculated by comparing out- and in- degree counts between node classes for 500 randomly permuted networks. Red line indicates empirical odds ratio.

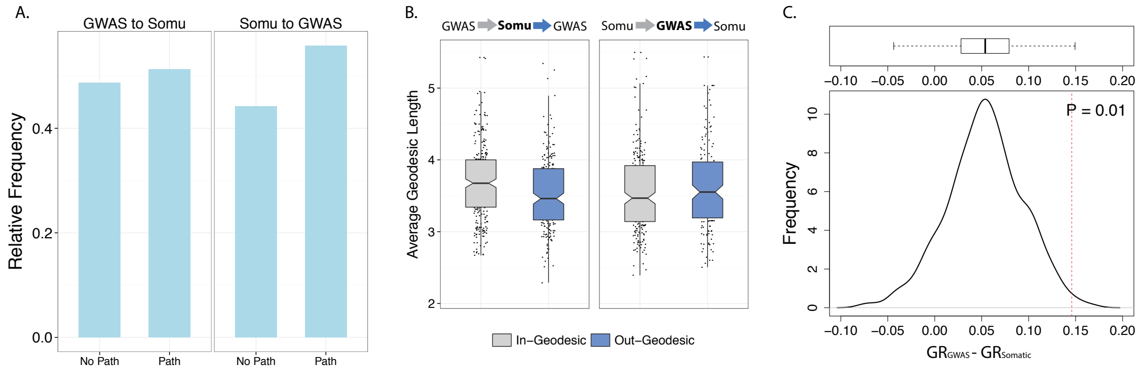

Figure S3. Comparison of geodesic ratios between GGNs and SMNs. a) Relative reachability between GGNs and SMNs. **b)** Comparison of out- and in- geodesic lengths between GGNs and SMNs. **c)** Null distribution of geodesic ratio differences between GGNs and SMNs.
